# Ultra-Flexible Organic Solar Cell Based on Indium-Zinc-Tin Oxide Transparent Electrode for Power Source of Wearable Devices

**DOI:** 10.3390/nano11102633

**Published:** 2021-10-07

**Authors:** Jun Young Choi, In Pyo Park, Soo Won Heo

**Affiliations:** Nanomaterials and Nanotechnology Center, Electronic Convergence Division, Korea Institute of Ceramic Engineering and Technology (KICET), 101, Soho-ro, Jinju-si 52851, Gyeongsangnam-do, Korea; ichoijy@naver.com (J.Y.C.); pyop4869@naver.com (I.P.P.)

**Keywords:** ultra-flexible organic solar cells, IZTO, transparent electrode, mechanical stability, 1D grating pattern

## Abstract

We have developed a novel structure of ultra-flexible organic photovoltaics (UFOPVs) for application as a power source for wearable devices with excellent biocompatibility and flexibility. Parylene was applied as an ultra-flexible substrate through chemical vapor deposition. Indium-zinc-tin oxide (IZTO) thin film was used as a transparent electrode. The sputtering target composed of 70 at.% In_2_O_3_-15 at.% ZnO-15 at.% SnO_2_ was used. It was fabricated at room temperature, using pulsed DC magnetron sputtering, with an amorphous structure. UFOPVs, in which a 1D grating pattern was introduced into the hole-transport and photoactive layers were fabricated, showed a 13.6% improvement (maximum power conversion efficiency (PCE): 8.35%) compared to the reference device, thereby minimizing reliance on the incident angle of the light. In addition, after 1000 compression/relaxation tests with a compression strain of 33%, the PCE of the UFOPVs maintained a maximum of 93.3% of their initial value.

## 1. Introduction

Recently, interest in human implantable sensors, skin-attached sensors, and wearable display devices that display acquired information in real time for fast and accurate monitoring of biosignals has been increasing [1,2,3,4]. When these devices are implanted or attached to the human body, they must be ultra-light-weight and flexible so that there is no heterogeneity on the skin or other human tissues [5,6]. Furthermore, it is also necessary to develop new device structures, such as ones with the sensor and power-supply units combined, as well as the development of power sources for the sensors [1,4,7]. Ultra-flexible organic photovoltaics (UFOPVs) are devices that convert light-energy into electrical energy. They are one of the important candidates for use as a power source for wearable devices such as biosensors [8,9,10,11]. UFOPVs exhibit a high signal-to-noise ratio because they have a lower noise current than batteries or conventional power supplies. Therefore, when applied to biosensors, improving the accuracy of the collection and analysis of biosignals is possible [1]. In addition, due to the thickness of UFOPVs not exceeding 5 μm, they have excellent adaptability to skin and other tissues given their flexibility and adhesion to 3D surfaces [12]. Therefore, in order to apply UFOPVs as power sources for wearable devices, such as implantable or attachable biosensors, the following conditions must be satisfied. First, the used transparent electrode must have excellent transmittance and conductivity, as well as mechanical flexibility [13]. Second, for the stable power supply, the dependence on incident light should be low [14]. Third, since various types of organic materials (substrate, photoactive layer, electrode, etc.) are used, the device manufacturing process is better at room temperature. Fourth, the fabricated device must have excellent mechanical stability [15,16]. Currently, indium-tin oxide (ITO) transparent electrodes are being applied to various optoelectronic devices, such as liquid crystal displays (LCDs), organic light-emitting diodes (OLEDs), organic photovoltaics (OPVs), and perovskite solar cells due to their excellent transmittance and conductivity [17,18,19,20]. However, in order to improve the electro-optical properties of ITO, a thermal treatment process of 300 °C or higher is required, which makes it difficult to apply to organic-based ultra-flexible substrates [21]. In addition, due to the brittle properties of ITO, cracks or fractures occur with only 2–3% deformation [22,23]. Therefore, the use of organic material-based transparent electrodes with excellent mechanical stability—such as poly (3,4-ethylenedioxythiophene): polystyrene sulfonate (PEDOT:PSS), metal nanowires or meshes, carbon nanotubes and graphene—is increasing [24,25,26,27]. However, due to their low conductivity, poor surface roughness, and complicated manufacturing process compared to ITO, further research on alternative materials to replace ITO is required. Therefore, in this study, we used parylene as an ultra-flexible substrate and amorphous indium-zinc-tin oxide (IZTO) as a transparent electrode, respectively, to fabricate UFOPVs as a power source that satisfies the various aforementioned conditions. Although the results of using IZTO for various purposes has been explored by other researchers, no study evaluating its mechanical properties using transparent electrodes for UFOPVs [28,29,30] had been done. Parylene has excellent optical transmittance (>90%), thermal stability (>290 °C), and chemical stability, making it suitable as an ultra-flexible substrate material [1,12,31]. The IZTO transparent electrode was fabricated using a sputtering target composed of 70 at.% In_2_O_3_-15 at.% ZnO-15 at.% SnO_2_ and deposited by pulsed DC magnetron sputtering system. The IZTO transparent electrode fabricated at room temperature showed an amorphous structure, a transmittance of 88.5% (including parylene substrate) in the visible wavelength region, and a mobility of 35 cm^2^/Vs. In addition, to reduce the dependence on the incident angle of light, soft imprinting lithography was applied to introduce a 1D grating pattern into the hole transport layer and the photoactive layer, respectively. All UFOPVs were fabricated at room temperature with conventional structures. After introducing the 1D grating pattern, the dependence of the incident angle of light in UFOPVs decreased by 2.2 times at 80°. In addition, to measure the mechanical stability of UFOPVs, they were attached to an elastomer film stretched by 200%, with compression/relaxation repeated 1000 times at 33% compressive strain. After the mechanical property test, the power conversion efficiency (PCE) of the UFOPVs using the IZTO transparent electrode showed a 30% improvement compared to a device using the ITO transparent electrode.

## 2. Materials and Methods

### 2.1. Materials

Parylene, an ultra-flexible substrate material, was purchased from KISCO Ltd. (diX-SR, Tokyo, Japan) and used without further purification. The composition of the IZTO target for transparent electrode was 70 at.% In_2_O_3_-15 at.% ZnO-15 at.% SnO_2_ and was purchased from Kojundo Chemical Laboratory Co., Ltd. (Saitama, Japan). For the PEDOT:PSS used as the hole transport layer, AI4083 (Ossila) was chosen. The electron donor polymer (PTB7) and electron acceptor material (PC_71_BM, >99%) were purchased from 1-material (Tokyo, Japan) and Solenne (Groningen, the Netherlands), respectively. Sylgard 184, a polydimethylsiloxane (PDMS) produced by Dow Corning (Midland, MI, USA), was used for the soft-patterned stamp.

### 2.2. Fabrication of 1D Grating Patterned PDMS

To fabricate a patterned PDMS stamp with a 1D grating structure, a blank DVD was used. We discussed in detail how to fabricate a patterned PDMS stamp in a previous study [8]. The polycarbonate protective layer of the blank DVD was removed using a surgical knife. When the pattern was exposed, the dye layer was removed with methanol, then dried out using a N_2_ gun. For the PDMS blend solution, a base material and curing agent were mixed in a weight ratio of 10:1, then degassed in a vacuum chamber. The prepared PDMS solution was poured onto the DVD and cured for 3 h on a hot plate at 90 °C. After the PDMS had solidified, it was peeled off from the DVD and used as the 1D grating patterned PDMS stamp. The period of the 1D grating pattern was 760 nm; the depth was 100 nm.

### 2.3. Preparation of Ultra-Flexible Substrate and IZTO Transparent Electrode

In order to easily delaminate parylene, an ultra-flexible substrate, from the sacrificial substrate, a self-assembly monolayer (SAM) for lowering the surface energy of the sacrificial substrate was formed. The sacrificial substrate was treated with UV/Ozone (Ahtech LTS Co., Ltd., Gyeonggi-do, Korea) for 30 min and a small Petri dish was placed in a container with a capacity of 300 mL. After that, 30 μL of trichloro(perfluorooctyl)silane (FOTS, 98%, Merck, Darmstadt, Germany) was dropped onto the Petri dish and the container lid was closed. It was then placed in a vacuum chamber of 5 × 10^−3^ Torr for 1 h. To fabricate a parylene ultra-flexible substrate, a SAM-treated sacrificial substrate was placed in a CVD chamber and filled with 10 g of parylene. A 1 μm ultra-flexible substrate was fabricated by its deposition in a CVD chamber of 5 × 10^−4^ Torr for 1 h. UVO treatment was performed for 10 min to improve the adhesion between the parylene and IZTO transparent electrode. The prepared substrate was loaded in a sputtering chamber. Sputtering was performed with 125 W input power, 30 kHz frequency, and 6 mTorr oxygen partial pressure, with a flow ratio of 3% [O_2_/(O_2_ + Ar)]. The deposition temperature was performed at room temperature. The thickness of the deposited IZTO film was 100 nm.

### 2.4. Fabrication of Ultra-Flexible OPVs

UFOPVs were fabricated with a 1D grating pattern applied to the hole transport layer (PEDOT:PSS), the photoactive layer, and again on both layers. For the device without patterns, 150 μL of PEDOT:PSS was applied and spin-coated at 3000 rpm for 30 s. Thermal annealing was performed on a hot plate at 140 °C for 20 min, with a fabricated thickness of 40 nm. In order to introduce a pattern to the PEDOT:PSS layer, 100 μL of PEDOT:PSS was applied and spin-coated at 5000 rpm for 10 s. The patterned PDMS was then quickly but gently placed on the coated PEDOT:PSS layer. After 60 s, the patterned PDMS stamp was removed and thermal annealed on a hot plate at 140 °C for 20 min. The period of the fabricated 1D grating pattern was 760 nm and the depth 35 nm. To form the photoactive layer, PTB7, as an electron donor material, and PC_71_BM, as an electron acceptor material, were used, dissolved in 1 mL of o-dichlorobenzene (DCB) at a weight ratio of 1:1.5 (10 mg:15 mg). Three volume percent of 1,8-diiodooctane (DIO, 98%, Merck) was added as a processing additive. The photoactive solution was spin-coated on a PEDOT:PSS layer, with or without a pattern, at 800 rpm for 7 s, then put in a N_2_-filled glove box. To form a nanostructure, the patterned PDMS was gently placed on the spin-coated photoactive layer, then removed after 60 s. Like the PEDOT:PSS layer, no pressure was applied to the patterned PDMS; even after the patterning process, the photoactive layer was not thermally treated. The period of the fabricated 1D grating pattern was 760 nm and the depth 55 nm. For the metal electrode, a thermal evaporator was used. A Ca electron transport layer (0.5 Å s^−1^, 20 nm) and an Al electrode (0.5 Å s^−1^, 100 nm) were deposited under a vacuum of 10^−7^ Torr, using a metal mask.

### 2.5. Measurements

The thicknesses of the prepared parylene thin film, IZTO, PEDOT:PSS, and photoactive layers were measured using a surface profiler (DEKTAK 6M, Bruker, Billerica, MA, USA). The transmittance of the IZTO transparent electrode was measured using UV-Vis spectroscopy (V-650, JASCO, Tokyo, Japan). The X-ray diffraction (XRD) pattern of the IZTO film was analyzed with a Rigaku SmartLab diffractometer. Monochromatic Cu Kα radiation (λ = 0.154 nm) was generated at 45 kV, 200 mA. The surface morphologies of the patterned PDMS, PEDOT:PSS, photoactive layer, and IZTO were observed using an AFM (5400 scanning probe microscope, Agilent Technologies, Santa Clara, CA, USA) in the tapping mode. The surface morphology of the IZTO film was characterized using a field emission scanning electron microscope (FESEM S-4800, Hitachi, Tokyo, Japan). Electrical properties, such as resistivity, carrier concentration, and mobility of the IZTO film, were measured using the van der Pauw method, together with a Hall measuring system (HMS-3000, Ecopia, Gyeonggi-do, Korea).

The UFOVPs’ current density-voltage characteristics were measured using a Keithley 2400 source meter unit under simulated solar illumination (AM 1.5, 100 mW cm^−2^) in a 150 W Xe lamp -based solar simulator (PEC-L11, Peccell Technologies, Yokohama, Japan). The light intensity was calibrated with a standard silicon solar cell (BS520, Bunkoh-Keiki, Tokyo, Japan). The active area of each device was defined, using a metal photomask, to be 0.12 cm^2^. The external quantum efficiency (EQE) of each device was measured with monochromatic light (SM-250F, Bunkoh-Keiki, Tokyo, Japan). For the mechanical stability test of the UFOPVs, they were removed from a sacrificial substrate and attached to a 200% pre-stretched acrylic–elastomer film (VHB Y-4905 J, 3M, Saint Paul, MN, USA). The degree of stretching was controlled using a home-made uniaxial screw machine and ruler, and the measurements were conducted under simulated solar illumination (AM 1.5, 100 mW cm^−2^) and ambient conditions. The pre-stretched acrylic elastomer film, to which the ultra-flexible OPV devices were attached, was slowly compressed in the uniaxial center direction, while reducing the stretching force. This mechanical test was conducted for 1000 repeated compression cycles, with a compression of 33%. The data obtained from the stretching test were recorded using a Keithley 2400 source meter unit.

## 3. Results and Discussion

**Figure 1:**
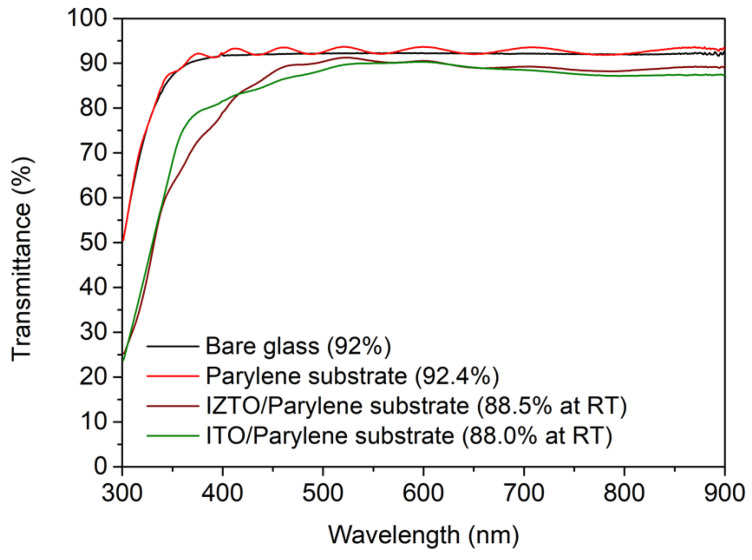
Transmission spectra of the bare glass, parylene free-standing film, the IZTO, and ITO films grown at room temperatures on the parylene substrates, respectively.

Figure 1 shows the UV-visible spectroscopy data of bare glass substrate, parylene ultra-flexible substrate, and IZTO transparent electrodes deposited at room temperature. IZTO was deposited on parylene at room temperature. The transmittance of the bare glass substrate in the visible wavelength region (380–780 nm) was 92%, whereas the transmittance of the parylene ultra-flexible substrate was 92.4%, which was slightly higher than bare glass substrate [32]. As can be seen in Figure 1, the spectra of the parylene substrate with the IZTO transparent electrode has a sinusoidal shape. This is because the parylene substrate is very thin (1 μm) and also because of the optical interference caused by the refractive index difference between the parylene substrate and air interface, or that of the parylene substrate and IZTO interface. An interesting point is that even without thermal treatment of the substrate, the IZTO/parylene transparent electrode showed a slightly higher transmittance of 88.5%, which was improved compared to the ITO/parylene transparent electrode (88%).

**Figure 2:**
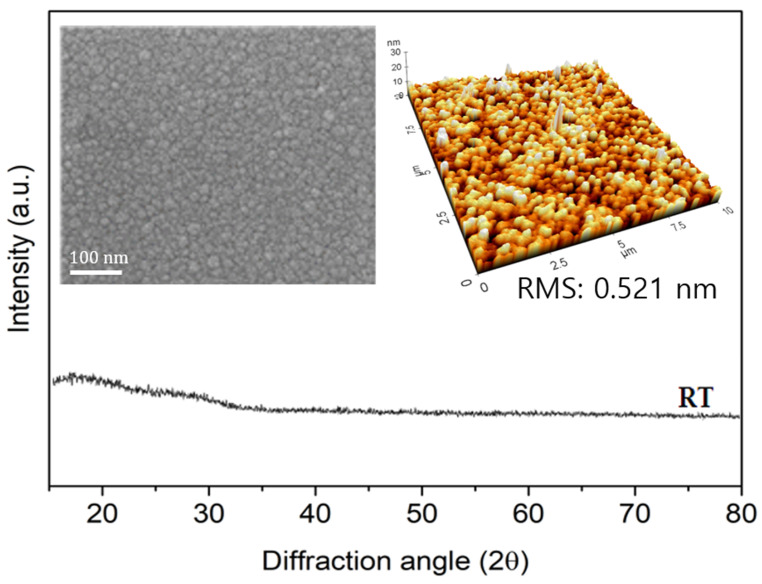
X-ray diffraction patterns of IZTO films with room temperature deposition on a parylene substrate. Insets are SEM image (**left**) and surface roughness (**right**) of IZTO film, respectively.

Figure 2 shows the XRD pattern of IZTO deposited at room temperature. The inset shows the surface morphologies of the IZTO thin film observed by FESEM and AFM. Combining the data in Figure 2, it can be seen that IZTO deposited at room temperature has an amorphous structure and a very low surface roughness of 0.521 nm. The resistivity, carrier concentration, and mobility of the IZTO thin film measured by the Hall measurement system were 6.2 × 10^−4^ Ω cm, 7.0 × 10^20^ cm^−3^, and 45 cm^2^ V^−1^ s^−1^, respectively, showing superior properties than the electrical properties of ITO fabricated at room temperature (resistivity: 8.3 × 10^−4^ Ω cm, carrier concentration: 5.3 × 10^20^ cm^−3^ and mobility: 35 cm^2^ V^−1^ s^−1^). Therefore, it was possible to conclude that the ultra-flexible transparent electrode based on IZTO can be used as the transparent electrode of a wearable device.

**Figure 3:**
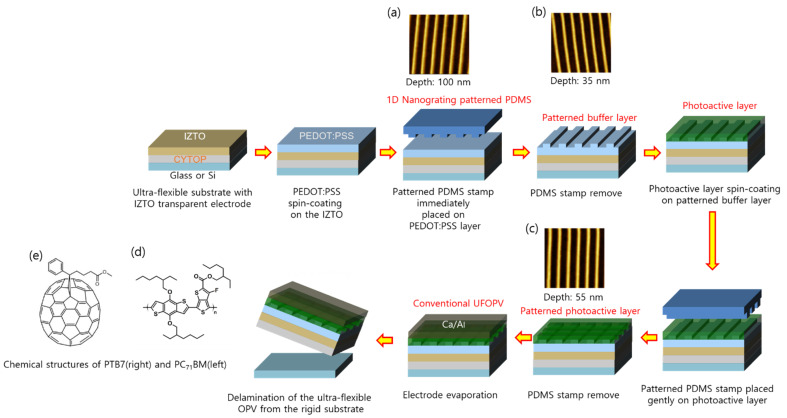
Schematic illustration of the fabrication of ultra-flexible OPVs with a 1D grating pattern by soft imprinting lithography using a PDMS mold. Inset figures present: (**a**) 1D grating patterned PDMS mold (period: 760 nm, depth: 100 nm); (**b**) 1D grating patterned PEDOT:PSS layer (period: 760 nm, depth: 35 nm); (**c**) 1D grating patterned photoactive layer; mixture of PTB7 and PC_71_BM (pitch: 760 nm, depth: 55 nm); (**d**,**e**), chemical structures of PTB7 and PC_71_BM, respectively.

The fabrication process of the UFOPVs—with the structure of IZTO/PEDOT:PSS (with pattern (p-PEDOT) and without (f-PEDOT), 40 nm)/PTB7:PC_7_1BM (with pattern (p-Al) and without (f-Al), 100 nm)/Ca (20 nm)/Al electrode (100 nm)—is shown in Figure 3. Nanostructures were introduced using a blank DVD template by applying soft imprinting lithography at room temperature. The surface morphology of the UFOPVs with nanostructures was characterized by AFM and is summarized in the inset of Figure 3a–c. The detailed IZTO-based UFOPVs’ fabrication method is described in Section 2.4. The 1D grating structure in the patterned PDMS stamp had a period of about 760 nm and a depth of 100 nm (Figure 3a). In PEDOT:PSS introduced through soft nanoimprinting lithography, the period of the 1D grating structure was the same as that of the PDMS stamp, and the depth was 35 nm (Figure 3b). Similarly, the period of the 1D grating structure in the photoactive layer was 760 nm and the depth 55 nm (Figure 3c). Figure 3d,e shows the chemical structures of the electron donor material and the electron acceptor material used in this study, respectively.

**Figure 4:**
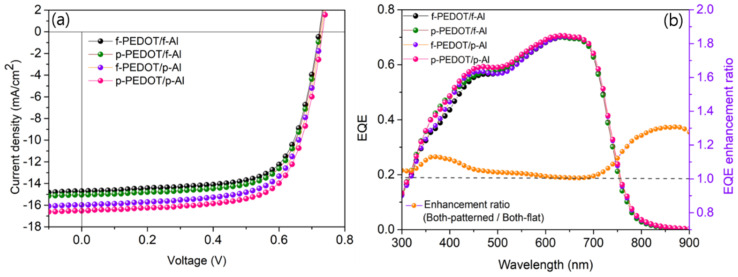
J-V characteristics of ultra-flexible OPVs based on PTB7:PC_71_BM BHJ with (**a**) f-PEDOT/f-Al, p-PEDOT/f-Al, f-PEDOT/p-Al, and p-PEDOT/p-Al. J-V characteristics of UFOPVs measured under AM 1.5G illumination at 100 mW cm^−2^. (**b**) EQE spectra and EQE enhancement ratios of the patterned devices relative to the reference.

Figure 4a shows the current density-voltage (J-V) characteristics of the IZTO-based UFOPVs. Detailed electrical properties, such as short circuit current density (J_SC_), open circuit voltage (V_OC_), fill factor (FF), and power conversion efficiency (PCE) are summarized in Table 1. The J_SC_ of the device in which the nanostructure was introduced into PEDOT:PSS (p-PEDOT/f-Al) was 15.08 mA cm^−2^, which was better than the reference device’s (f-PEDOT/f-Al) 14.83 mA cm^−2^. In addition, the FF of the p-PEDOT/f-Al structure device was 69.3%, slightly better than the reference device (68.0%). All V_OC_ were 0.72 V, with no significant change. With the increase of the J_SC_ and FF, the PCE of the device of the p-PEDOT/f-Al structure was improved by 3.6% from 7.26% (reference device) to 7.52%. When a 1D grating pattern was introduced into the photoactive layer (f-PEDOT/p-Al), the J_SC_ value increased to 16.05 mA cm^−2^, with ignorable change in the V_OC_ and FF, and the calculated PCE showing 7.95%. In addition, when 1D grating patterns were introduced in both the PEDOT:PSS and photoactive layer (p-PEDOT/p-Al), V_OC_ stayed at 0.72 V, while J_SC_ increased to 16.81 mA cm^−2^, showing the highest PCE of 8.35%.

As can be seen in Table 1, when the 1D grating pattern was introduced into both the PEDOT:PSS and the photoactive layer, the series resistance (R_S_) and shunt resistance (R_SH_) values both improved compared to the reference device. The FF and J_SC_ increased by 1.5% and 13.4%, respectively. In addition, when the 1D grating pattern was introduced into the PEDOT:PSS layer, transmittance in the 300–450 nm wavelength region was improved due to the anti-reflection effect. Figure 4b shows that the EQE value in the 300–450 nm wavelength region was better than the reference device. Therefore, it can be seen that the introduction of nanostructures in the PEDOT:PSS layer leads to the improvement of J_SC_. Furthermore, the introduction of a 1D grating pattern into the photoactive layer significantly increased the J_SC_ to 16.05 mA cm^−2^ (p-PEDOT/f-Al) and 16.81 mA cm^−2^ (p-PEDOT/p-Al), respectively. In addition, propagating surface plasmon polariton (p-SPP), generated at the patterned photoactive layer and metal interface, also induces the cumulative gain of J_SC_. This can be confirmed by observing the wavelength region after 700 nm in Figure 4b. Finally, when a 1D grating pattern was introduced in all layers, J_SC_ increased to 16.81 mA cm^−2^, showing the highest PCE of 8.35%, due to the combined effects of anti-reflection, scattering, and propagating surface plasmon polariton.

**Figure 5:**
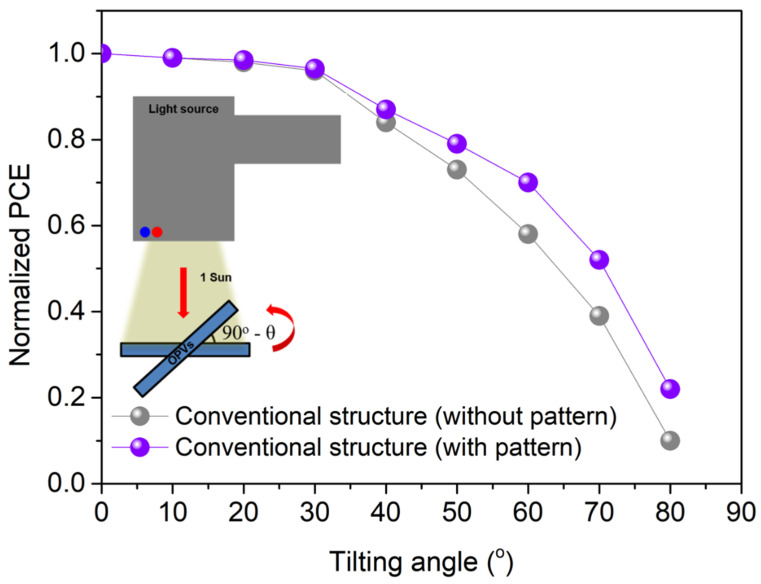
Incident angle dependence characteristics of with/without patterned ultra-flexible OPVs. Data shows normalized values of PCE. Inset shows the illustration of incident angle of light.

Figure 5 shows the data analyzing the PCE characteristics of the UFOPVs with respect to the incident angle of light. It was confirmed that the device in which the nanostructure was introduced had a reduced dependence on incident light compared to the device without the nanostructure. This is because the incident light is scattered by the 1D grating pattern formed between the PEDOT:PSS layer and the photoactive layer, thereby increasing the optical path length. Therefore, the absorption amount of photons at a low incident angle is increased compared to a device without a nanostructure. When the incident angle was 80°, the PCE of UFOPVs with nanostructures was improved by 2.2 times compared to the reference device. This demonstrates that UFOPVs with nanostructures can be used as power sources for wearable devices.

**Figure 6:**
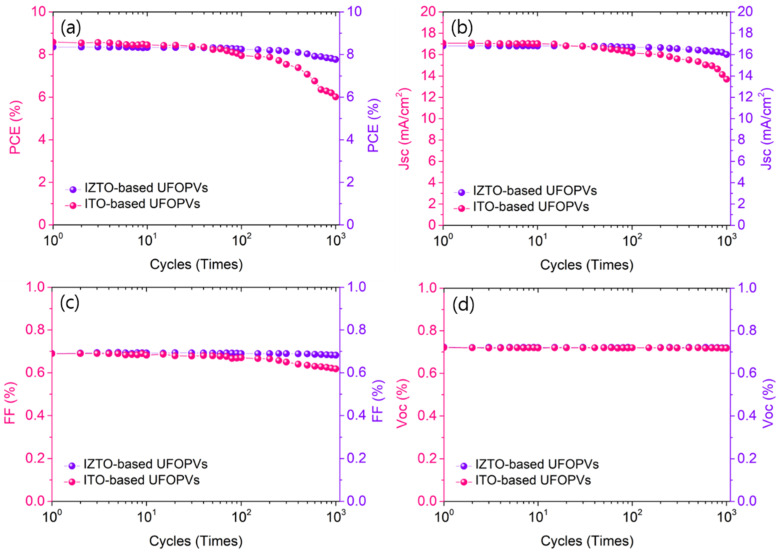
Behavior of photovoltaic parameters under cyclic compression of ultra-flexible OPVs with ITO, and ultra-flexible OPVs with IZTO: (**a**) PCE, (**b**) J_SC,_ (**c**) FF, and (**d**) Voc.

To confirm the mechanical stability of IZTO/parylene-based UFOPVs, repeated compression tests were performed. The UFOPVs delaminated from the sacrificial substrate were attached to a 200% pre-stretched acrylic elastomer film. UFOPVs fixed on an elastomer film were compressed in a uniaxial direction. Compression and relaxation were repeated 1000 times at a compression strain of 33% and their results are summarized in Table 2. In the case of IZTO-based UFOPVs, the PCE decreased by 6.7%, from 8.3% to 7.8%. On the other hand, in the case of UFOPVs using ITO as a transparent electrode, the PCE decreased by 43%, from 8.6% to 6%. As can be seen in Figure 6a–c, the PCE of the ITO-based device decreased due to the reduction of J_SC_ and FF. On the other hand, the decrease in J_SC_ and FF was not noticeable in the IZTO-based device due to the high brittleness of ITO. It was confirmed that the amorphous IZTO showed excellent mechanical stability despite the test’s harsh conditions. In addition, by using flexible PEDOT:PSS as a hole transport layer, it is expected that PEDOT:PSS would resist deformation, thereby playing a role in protecting the IZTO transparent electrode.

## 4. Conclusions

We successfully fabricated 1 μm-thick UFOPVs that were highly compatible with the human body and capable of supplying stable energy. The substrate of UFOPVs used parylene, and the IZTO used as the transparent electrode was fabricated at room temperature through pulsed DC magnetron sputtering system using a sputtering target composed of 70 at.% In_2_O_3_-15 at.% ZnO-15 at.% SnO_2_. The 1 μm-thick parylene, ultra-flexible substrate showed a high transmittance of 92.4%. The IZTO/parylene-based transparent electrode, to which IZTO deposited at room temperature was applied, has an amorphous structure and a demonstrated transmittance of 88.5%. In addition, the resistivity, carrier concentration, and mobility were 6.2 × 10^−4^ Ω cm, 5.3 × 10^20^ cm^−3^, and 35 cm^2^ V^−1^ s^−1^, respectively.

In addition, we introduced a 1D grating pattern on the hole transport and photoactive layers through soft imprinting lithography at room temperature to lower the dependence on the incident angle of light and thereby obtain higher output power. The fabricated device showed a PCE of 8.35%, which is an improvement of 13.6% compared to the reference device. With the introduction of the 1D grating pattern, anti-reflection, scattering, and propagation surface plasmon polaritons were expressed in a complex manner, causing the cumulative accumulation of J_SC_. Moreover, with the introduction of the 1D grating pattern, the dependence on the incident angle of light was reduced by 2.2 times at 80°. IZTO/parylene-based UFOPVs also showed very good mechanical stability. Although compression–relaxation was repeated 1000 times with a compression strain of 33%, there was only a 6.7% decrease compared to the initial PCE. Therefore, IZTO/parylene-based, ultra-flexible, transparent electrodes can be used for future optoelectronic devices. Furthermore, fabricated UFOPVs can be combined with various wearable devices, as well as used self-powered devices that do not require an external power source.

## Figures and Tables

**Table 1:** Device characteristics of various device structures. The average values were calculated from eight or more cells. R_SH_ and R_S_ were obtained from the slope of the J-V curves in the dark at 0 and 1 V, respectively.

Device Structure		J_SC_ (mA cm^−2^)	V_OC_ (V)	FF (%)	PCE (%)	Increment in PCE (%)	R_SH_ (kΩ cm^2^)	R_S_ (Ω cm^2^)
IZTO	Reference (f-PEDOT/f-Al)	14.83 ± 0.02	0.72	68.0 ± 0.2	7.26 ± 0.3	-	4.1	3.51
	p-PEDOT/f-Al	15.08 ± 0.06	0.72	69.3 ± 0.3	7.52 ± 0.2	3.6	9.9	2.78
	f-PEDOT/p-Al	16.05 ± 0.04	0.72	68.8 ± 0.2	7.95 ± 0.4	8.1	9.0	2.74
	p-PEDOT/p-Al	16.81 ± 0.06	0.72	69.0 ± 0.2	8.34 ± 0.3	13.6	15.3	2.32
ITO	p-PEDOT/p-Al	17.07 ± 0.03	0.72	68.9 ± 0.3	8.58 ± 0.2	-	15.8	2.22

**Table 2:** Device characteristics of various device structures after mechanical test.

Device Structure		J_SC_ (mA cm^−2^)	V_OC_ (V)	FF (%)	PCE (%)
After mechanical test	ITO	13.69	0.71	61.8	6.01
	IZTO	16.01	0.71	68.2	7.75

## Data Availability

The data presented in this study are available on request from the corresponding author.
